# Supplementary material for: Identification of celastrol as a novel HIV-1 latency reversal agent by an image-based screen
Source: PLoS One. 2021 Apr 29;16(4):e0244771. doi: 10.1371/journal.pone.0244771 (PMC8084242; doi:10.1371/journal.pone.0244771)

Non-manipulated image Figure 5 left panel

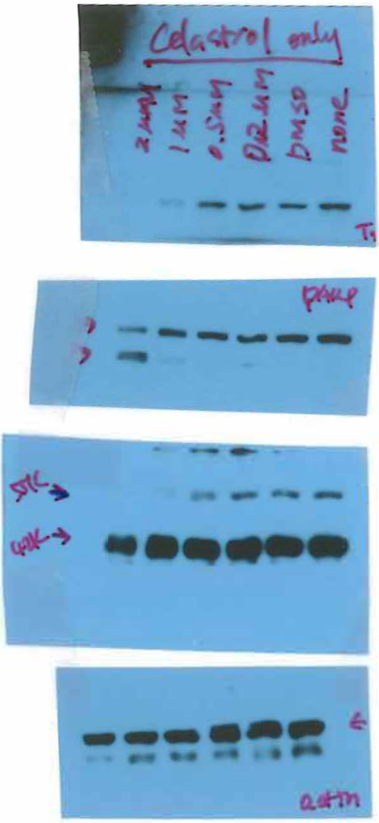

Non-manipulated image Figure 5 middle panel

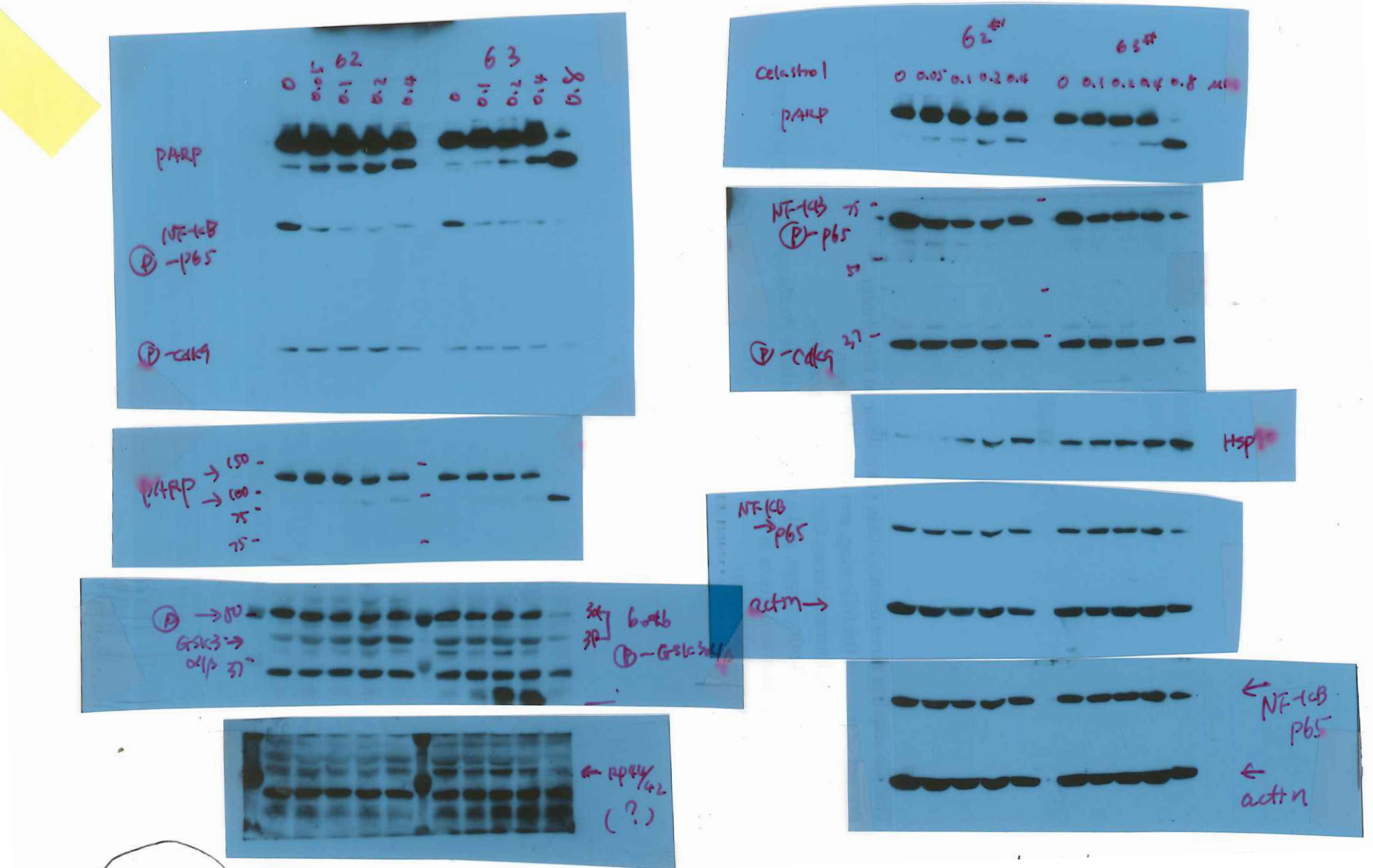

Non-manipulate image Figure 5 right panel

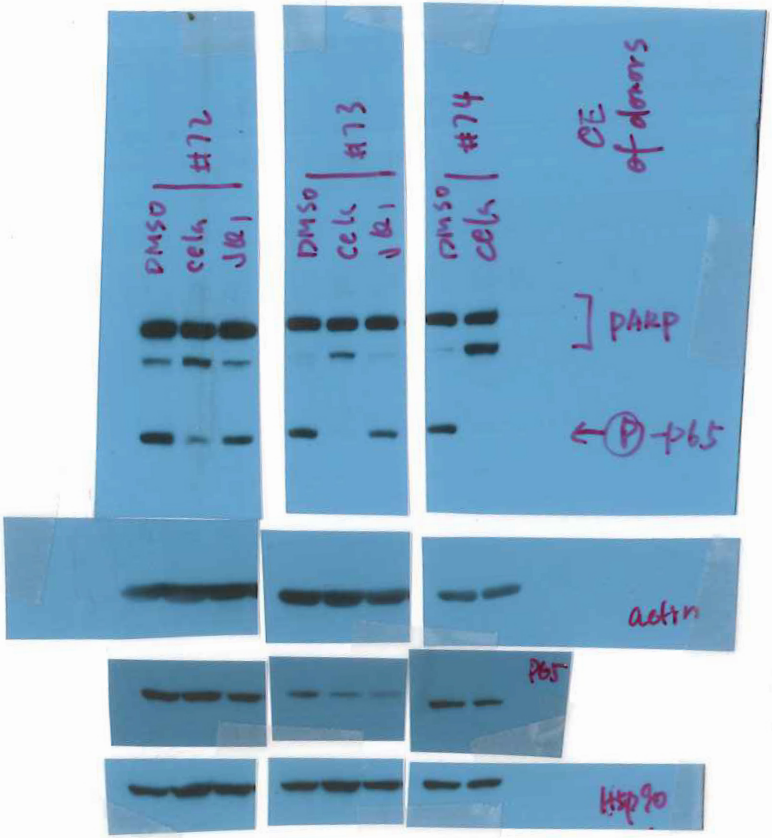

Rabbit-
αNFKB p65. sc-109 Santa Cruze

phospho-NF-κB-p65 3033S. Cell Signaling
mouse

Non-manipuated image Figure 7

3/9/2021 To revize paper 1st try

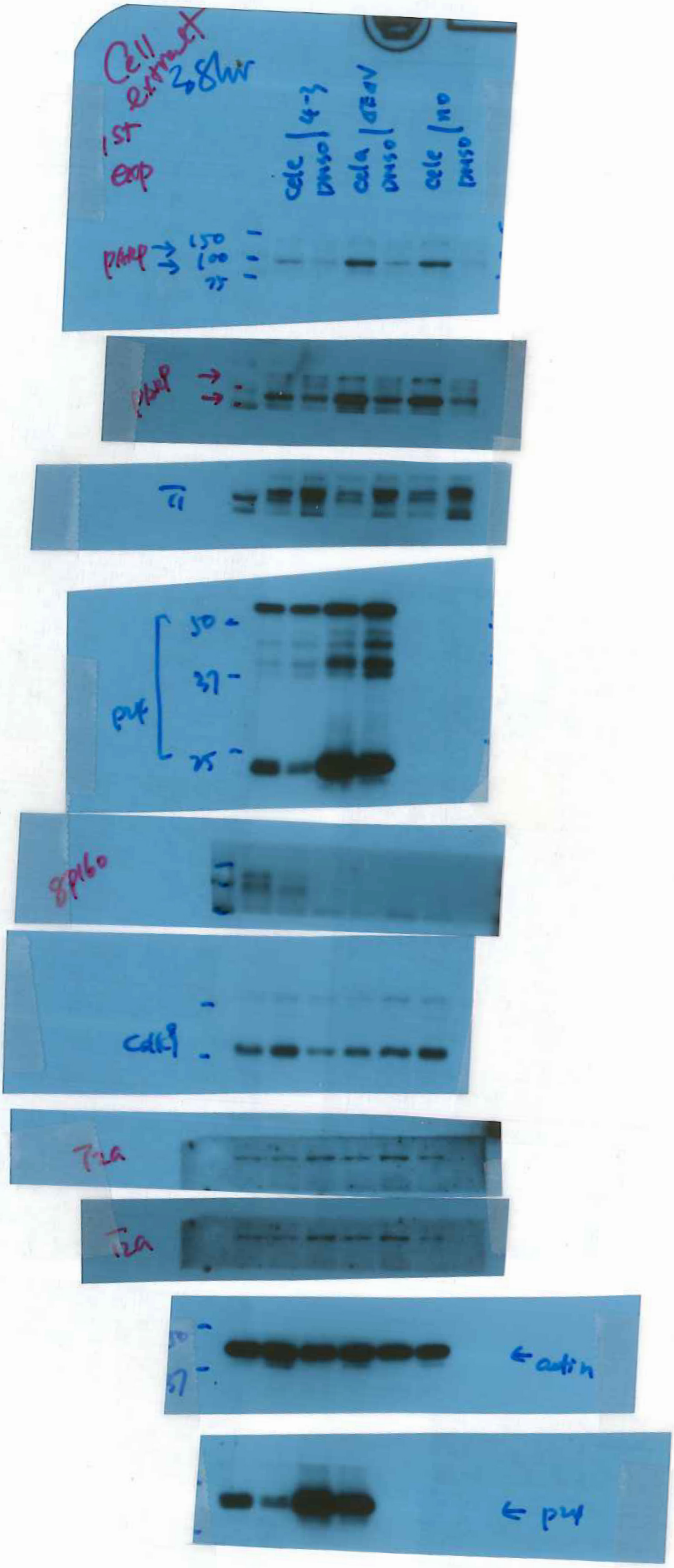

Supplement: S1 Raw images — (PDF) [file pone.0244771.s001.pdf]
