# Supplementary material for: Identification of celastrol as a novel HIV-1 latency reversal agent by an image-based screen
Source: PLoS One. 2021 Apr 29;16(4):e0244771. doi: 10.1371/journal.pone.0244771 (PMC8084242; doi:10.1371/journal.pone.0244771)
Supplement: Attachment — Submitted filename: Comments-Liu et. al.pdf [file pone.0244771.s002.pdf]

In this publication by Liu et al, has found a new class of HIV-1 Latency Reversing Agent (LRA), namely celastrol, following the screening of a natural compound library. The authors very convincingly demonstrated that celastrol quite selectively stimulates latently infected T cells, but not much to the uninfected resting primary CD4+ T cells. However, the precise underlying mechanism through which celastrol works is under investigation. The Rice lab has contributed enormously to the field of HIV transcription and latency, by doing a number of seminal studies. This publication marks the first set of results, which are very exciting and groundbreaking. In this investigation, for disrupting as minimal as possible the cellular state during experimentation, authors used a novel image-based assay.

Overall, this is a very well controlled and organized ongoing study. In this manuscript authors have presented their initial results. Authors convincingly demonstrate the strong synergistic activity of celastrol with other LRAs, however, defining of the underlying molecular mechanism is still under investigation.

Major Concern:

- Given the fact that celastrol synergizes with other LRAs in reactivating latent HIV, it will be interesting to check if celastrol enhances the cell/nuclear translocation of drugs/factors.
- Interestingly, celastrol inhibits NF-kB, and reduces Cyclin T1 levels, but still able to enhance HIV transcription, indeed directing towards a novel mechanism for HIV transcription. I will be crucial to show if celastrol also enhance HIV replication in your system.

Minor Concern:

- Please clearly indicate which NF-kB (p65) residue was assessed for determining the NF-kB phosphorylation.
- Line 75 of page-5 needs editing, maybe they want to say" An important property of any LRA is the absence of significant resting T cell activation, an activity that may induce systemic inflammation".
- Line 294 of page-14 needs editing, maybe authors want to add inhibition in the sentence "Additionally, celastrol has been reported to INHIBIT HIV-1 Tat activation of viral gene expression directed by the viral LTR in the U937 cell line [35]".
