# Supplementary material for: Identification of celastrol as a novel HIV-1 latency reversal agent by an image-based screen
Source: PLoS One. 2021 Apr 29;16(4):e0244771. doi: 10.1371/journal.pone.0244771 (PMC8084242; doi:10.1371/journal.pone.0244771)
Supplement: Attachment — Submitted filename: Response to Review.docx [file pone.0244771.s003.docx]

**Response to review**

We thank the Reviewer for positive comments and useful critiques that have improve our manuscript. Our response to each concern are:

**Major Concern:**

- *Given the fact that celastrol synergizes with other LRAs in reactivating latent HIV, it will be interesting to check if celastrol enhances the cell/nuclear translocation of drugs/factors.*

Response: This is an interesting suggestion and may provide mechanistic insight. However, we believe that the scope of such an analysis is quite broad and would merit its own publication.

- *Interestingly, celastrol inhibits NF-kB, and reduces Cyclin T1 levels, but still able to enhance HIV transcription, indeed directing towards a novel mechanism for HIV transcription. I will be crucial to show if celastrol also enhance HIV replication in your system.*

Response: We thank the reviewer for suggesting this experiment and we have performed it. The new data are presented in new Figure 7. Jurkat cells were infected with either HIV-1 NL4-3 or HIV-1 NL4-3Δenv/Vpr and intracellular p24 was quantified in immunoblots. The entire experiment presented in Figure 7 was repeated, so that we have analyzed four independent infections. The data indicate that celastrol modesty enhances p24 expression during infection, despite reductions in Cyclin T1 and CDK9 levels (Tat co-factors). This agrees with data presented in Figure 3 that celastrol functions in a Tat-independent mechanism.

**Minor Concern:**

- *Please clearly indicate which NF-kB (p65) residue was assessed for determining the NF-kB phosphorylation.*

Response: We have indicated in the revised manuscript in the Antibodies section of Methods and in the Results (line 252) section that this phosphorylated residue is Ser536.

- *Line 75 of page-5 needs editing, maybe they want to say” An important property of any LRA is the absence of significant resting T cell activation, an activity that may induce systemic inflammation”.*

Response: We have made this suggested edit in the revised manuscript.

- *Line 294 of page-14 needs editing, maybe authors want to add inhibition in the sentence “Additionally, celastrol has been reported to INHIBIT HIV-1 Tat activation of viral gene expression directed by the viral LTR in the U937 cell line [35]”.*
- Response: We have made this suggested edit in the revised manuscript.
